# Whole-Genome Sequence of Erwinia persicina B64, Which Causes Pink Soft Rot in Onions

**DOI:** 10.1128/MRA.01302-18

**Published:** 2019-01-03

**Authors:** Heejung Cho, Ji Yeon Park, Yong Ki Kim, Seong-Han Sohn, Dong Suk Park, Young-Seok Kwon, Cheol-Woo Kim, Chang-Gi Back

**Affiliations:** a National Institute of Agricultural Sciences, Rural Development Administration, Jeonju, Republic of Korea; b National Institute of Horticultural and Herbal Science, Rural Development Administration, Wanju, Republic of Korea; Louisiana State University

## Abstract

Erwinia persicina B64 was isolated from rotten onions in cold-storage facilities. Here, we report the complete genome sequence of E. persicina B64, which contains 5,070,450 bp with 55.17% GC content. The genome of this isolate is composed of one chromosome and two plasmids.

## ANNOUNCEMENT

Erwinia persicina is a rod-shaped Gram-negative bacterium that belongs to the Enterobacteriaceae family of the *Gammaproteobacteria* class. The bacterium has been reported to be phytopathogenic in various grain legumes (Phaseolus vulgaris, Medicago sativa, Glycine max, and Pisum sativum), causing necrotic spots on leaves (1–3) and causing a pinkish rot in garlic bulbs (Allium sativum L.) (4). It was previously reported that the causative agents of soft rot in stored onions (Allium cepa L.) were Erwinia rhapontici, Burkholderia cepacia, Pseudomonas marginalis, and Pseudomonas aeruginosa (5–7). This is the first report of E. persicina isolated from onion soft rot.

E. persicina B64 (Korean Agricultural Culture Collection deposit number 19353) was isolated from a rotten onion that was stored for nine months in cold storage. Onion is an important vegetable worldwide, with 117 million tons produced in 2016 (FAOSTAT [http://www.fao.org/faostat/en/#data]). In South Korea, this crop is important as a seasoning vegetable, and it is harvested in June and stored until the following April in cold-storage facilities. Stored onions deteriorate due to physiological and pathological disorders, resulting in a loss of 15 to 35% by the end of the storage period (8, 9).

**FIG 1:**
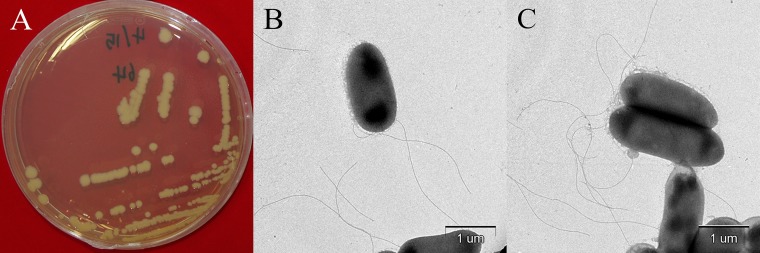
Erwinia persicina B64. Colonies on LB agar (A) and transmission electron micrographs (B and C).

In March 2016, rotten onions were obtained from a storage warehouse of NH Seed Research & Development Center, located in Yeongam, Jeollanam-do, Republic of Korea. From onions with soft rot, we isolated pathogens by excising the lesions, sterilizing the surface with 70% ethanol, washing with distilled water, loading the samples onto potato dextrose agar, and incubating at 25°C. Isolate B64, causal agent of pink soft rot in onion, was identified using 16S rRNA gene sequence homology, which was carried out by a BLAST search at the National Center for Biotechnology Information (NCBI) website (https://blast.ncbi.nlm.nih.gov/Blast.cgi), and using average nucleotide identity (ANI) values, which were calculated by uploading two genome FASTA files on the EZBioCloud website (https://www.ezbiocloud.net/tools/ani). For the results, B64 was identified to E. persicina based on 99% identity of 16S rRNA gene sequence homology and 99.44% ANI values of the whole genome of the B64 isolate with E. persicina NBRC 102418 (NCBI RefSeq accession number NR_114078). Observing the E. persicina B64 morphology under a transmission electron microscope (LEO 912AB; Carl Zeiss Co. Ltd., Germany) using negative staining with 0.5% uranyl acetate (10) revealed a rod-shaped appearance with one or more flagella and protruding vesicles in some bacteria (Fig. 1).

For whole-genome sequencing, E. persicina B64 was cultured in lysogeny broth (LB) in a shaker at 28°C, and 1 ml of culture at an optical density at 600 nm (OD_600_) of 0.7 was harvested and centrifuged at 13,000 rpm for 1 min. Genomic DNA was prepared using the Wizard SV genomic DNA purification system (Promega, USA), according to the manufacturer’s instructions. We sequenced the isolate B64 using a PacBio single-molecule real-time (SMRT) sequencing technology RS II system (Pacific Biosciences, USA) with a 20-kb library and P6-C4 chemistry. We produced 127,121 reads containing a total of 1,167,341,905 bp of 230-fold sequencing depth and an *N*_50_ length of 19,069 bp. *De novo* assembly was performed using the PacBio SMRT Analysis 2.3.0 HGAP.2 software, with a minimum seed length of 6,000 bp, conducted by ChunLab, Inc. (South Korea); it has been reported that HGAP does not require PacBio raw read error correction with short reads due to the intrinsic function of the generation of highly accurate long sequences (11).

The complete genome of E. persicina B64 had a total size of 5,070,450 bp, GC content of 55.17%, 4,774 coding sequences, 22 rRNAs, and 82 tRNAs (Table 1). The genome is composed of three circular contigs, with one circular chromosome of 4,795,673 bp and two circular plasmids of 144,252 bp (pEP1) and 130,525 bp (pEP2). This is the first report of the complete genome sequence of E. persicina, which is the first pathogen discovered from pink soft rot in onions.

**TABLE 1:** General genome features of Erwinia persicina strain B64

Feature	*E*. *persicina* B64
Genome size (bp)	5,070,450
GC content (%)	55.17
*N*_50_ (bp)	4,795,673
No. of coding sequences	4,774
No. of rRNAs	22
No. of tRNAs	82
No. of contigs	3
GenBank accession no.	CP022725, CP022726, CP022727

### Data availability.

The genome sequence and raw read data of E. persicina B64 were deposited at the National Center for Biotechnology (NCBI, USA) GenBank database under the accession numbers CP022725, CP022726, and CP022727 and SRA accession number SRR5937954.
